# Temperature-induced reversible self-assembly of diphenylalanine peptide and the structural transition from organogel to crystalline nanowires

**DOI:** 10.1186/1556-276X-9-653

**Published:** 2014-12-03

**Authors:** Renliang Huang, Yuefei Wang, Wei Qi, Rongxin Su, Zhimin He

**Affiliations:** 1 School of Environmental Science and Engineering, Tianjin University, Tianjin 300072, People’s Republic of China; 2 State Key Laboratory of Chemical Engineering, School of Chemical Engineering and Technology, Tianjin University, Tianjin 300072, People’s Republic of China; 3 Collaborative Innovation Center of Chemical Science and Engineering (Tianjin), Tianjin 300072, People’s Republic of China

**Keywords:** Self-assembly, Diphenylalanine, Peptide, Nanowire, Organogel

## Abstract

Controlling the self-assembly of diphenylalanine peptide (FF) into various nanoarchitectures has received great amounts of attention in recent years. Here, we report the temperature-induced reversible self-assembly of diphenylalanine peptide to microtubes, nanowires, or organogel in different solvents. We also find that the organogel in isopropanol transforms into crystalline flakes or nanowires when the temperature increases. The reversible self-assembly in polar solvents may be mainly controlled by electronic and aromatic interactions between the FF molecules themselves, which is associated with the dissociation equilibrium and significantly influenced by temperature. We found that the organogel in the isopropanol solvent made a unique transition to crystalline structures, a process that is driven by temperature and may be kinetically controlled. During the heating-cooling process, FF preferentially self-assembles to metastable nanofibers and organogel. They further transform to thermodynamically stable crystal structures via molecular rearrangement after introducing an external energy, such as the increasing temperature used in this study. The strategy demonstrated in this study provides an efficient way to controllably fabricate smart, temperature-responsive peptide nanomaterials and enriches the understanding of the growth mechanism of diphenylalanine peptide nanostructures.

## Background

Supramolecular self-assembly, a ubiquitously spontaneous process in nature, plays an important role in building highly ordered and functional structures in biology. It also provides a powerful tool for creating supramolecular nanostructures for various applications [1-5]. A substantial amount of research has focused on this issue, and various artificial self-assembling systems have been developed for peptides [1-4], peptide amphiphiles [5-7], and aromatic small molecules [8-10]. In particular, peptide-based supramolecular nanostructures have received great attention in recent years due to their excellent biocompatibility and functional diversity. Therefore, many peptide-based building blocks, including aromatic dipeptides [1,4,11-14], surfactant-like peptides [15], amyloid peptide fragments [16,17], and cyclic peptides [3], have been designed and developed for the construction of organized supramolecular nanostructures. Among these peptide-based self-assembling molecules, diphenylalanine peptide (l-Phe-l-Phe, FF), which is the core recognition motif of Alzheimer's Aβ peptides, is a fascinating unit that can self-assemble into diverse structures, such as microtubes, nanowires, and microcrystals. These assembled nanomaterials are extremely attractive as potential building blocks in the field of sensors, imaging, nanofabrication, and so on [1,4,18-21].

Controlling the self-assembly of small molecules into highly ordered architectures is an interesting topic in supramolecular chemistry. For the diphenylalanine peptide, the use of different organic solvents is employed to control the self-assembly behavior. For instance, diphenylalanine self-assembles to hollow tubular structures in aqueous or methanol solutions [22-26]. A structural transition from FF microtubes to highly uniform nanowires was demonstrated in our previous study by introducing acetonitrile as a cosolvent in water [27]. Interestingly, diphenylalanine also self-assembles in toluene or chloroform and forms amorphous nanofibers (gel), which are significantly different from the crystalline nanowires previously mentioned [19]. Another control strategy is the addition of a surface in the assembly solution, such as glass [27,28], or porous membrane [28]. The coexistence of the solvent and the surface makes controlling the morphologies of the peptide assemblies easier and more efficient. In these studies, the self-assembly of diphenylalanine peptides generally occurred at room temperature. Previously, Heredia et al. [29] demonstrated an irreversible phase transition of FF nanotubes from the hexagonal phase to another (most likely orthorhombic) crystalline phase at approximately 140°C to 150°C, indicating that temperature may be an important factor for controlling the structures of FF assemblies; however, the effect of temperature on the self-assembly of FF is often neglected. This study aims to develop a temperature-based control method for the self-assembly of diphenylalanine.

Temperature was considered an important parameter in the vapor-assisted self-assembly of diphenylalanine, in which a high temperature (e.g.,150°C, 220°C) was required for the structural transition or evaporation of FF molecules. For example, Ryu and Park reported a high temperature (150°C) aniline vapor aging process to synthesize a well-aligned peptide nanowire array starting from an amorphous peptide thin film [30]. By increasing the temperature to 250°C, the FF molecules evaporated and self-assembled on a substrate to form single crystalline nanowires [31]. These previous studies focus on the vapor-assisted self-assembly at high temperature; however, it is still unclear how the temperature (especially, temperatures lower than 100°C) influences the self-assembly of diphenylalanine in solvents or the structures of assemblies.

Herein, we report the temperature-induced reversible self-assembly of diphenylalanine peptide in solvents. Different solvents, including acetonitrile, acetonitrile-H_2_O, H_2_O, HFIP-H_2_O, and isopropanol, were chosen to demonstrate the assembly behavior. A temperature-responsive FF organogel in isopropanol is outlined. In this system, we further demonstrate a temperature-induced structural transition from an organogel to a crystalline flake-like structure in isopropanol. Additionally, the nanofibers in the organogel transferred into uniform crystalline nanowires with the assistance of a glass surface at an increased temperature. This temperature-based control process and the results of this study provide an efficient method for controlling the self-assembly of diphenylalanine peptide.

## Methods

### Materials

The lyophilized diphenylalanine peptide (NH_2_-Phe-Phe-COOH, FF) was purchased from Bachem (Bubendorf, Switzerland). The 1,1,1,3,3,3-hexafluoro-2-propanol (HFIP) was bought from Sigma-Aldrich (St. Louis, MO, USA). Acetonitrile and isopropanol were obtained from Aladdin Industrial Co. (Shanghai, China). All other reagents were of the highest grades available commercially.

### Temperature-induced self-assembly of FF in different solvents

Suspensions were prepared using 2 mg diphenylalanine peptide added to 1 mL each of acetonitrile, acetonitrile-water (95:5 *v*/*v*), pure water (H_2_O), or isopropanol. The resulting suspensions were then heated to 90°C for 5 min, forming a transparent solution. The FF nanowires, microtubes, or transparent gels were formed during cooling to ambient temperature (25°C) without any disturbance.

### Temperature-induced reversible self-assembly

After the formation of FF assemblies following the procedure mentioned above, the solution or gel containing FF assemblies was heated to 90°C for 5 min again. The assemblies were disassembled leading to the formation of a transparent solution. Then, the solution was cooled to 25°C without any disturbance. The disassembled FF molecules further self-assembled to nanowires, microtubes, or an organogel in different solvents again. All the heating-cooling experiments were carried out in the sealed tubes to prevent the volatilization of solvents. Three heating-cooling cycles were operated to confirm this reversible self-assembly process.

### Temperature-induced structural transition in isopropanol

The FF organogel was stored at either -25°C for over 2 months or 25°C for 3 days. Next, 50 μL FF gel was deposited onto a microscopic glass coverslip and sequentially air-dried at -25°C and 25°C, respectively. The resulting samples were then observed by scanning electron microscopy.

### Scanning electron microscopy

All the samples were sputter coated with platinum using an E1045 Pt-coater (Hitachi, Tokyo, Japan) and then imaged by using an S-4800 field emission scanning electron microscope (Hitachi High-Technologies Co., Tokyo, Japan) at an acceleration voltage of 5 kV. To observe the real structure of the organogel, the sample was prepared by flash freezing with liquid nitrogen followed by air drying at -45°C.

### X-ray diffraction

X-ray diffraction measurements were performed on a D8 Focus powder diffractometer (Bruker, Karlsruhe, Germany). The diffraction intensity of CuKa radiation (wavelength of 1.5418 nm) was measured under 40 kV and 40 mA with a scan rate of 2°/min in a 2*θ* range between 3° and 50°.

### Size distribution measurement

The size distribution and mean diameter, *d*, of FF assemblies in HFIP-water were determined using a Zetasizer Nano (0.4 nm to 10 μm, Malvern Instruments, Worcestershire, UK) particle size analyzer. The FF self-assembly was performed in a quartz cell directly following the heating-cooling procedure. The initial temperature was set at 90°C, and then, the samples were cooled to 80°C, 70°C, 60°C, 50°C, 40°C, and 30°C. All the temperature points were maintained for 30 min to ensure the complete self-assembly of the FF. The experimental analysis was repeated three times.

## Results and discussion

**Figure 1:**
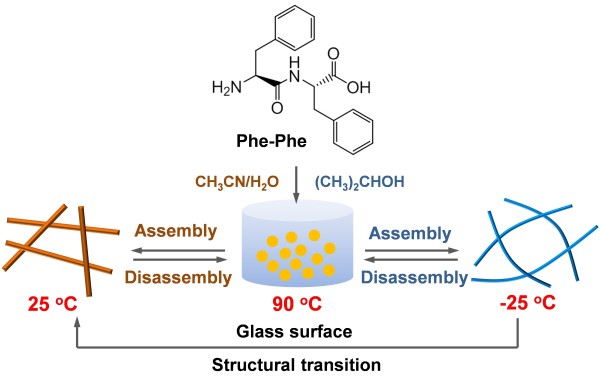
**Schematic illustration.** The temperature-induced reversible self-assembly of diphenylalanine peptide and the structural transition from organogel to crystalline nanowires.

**Figure 2:**
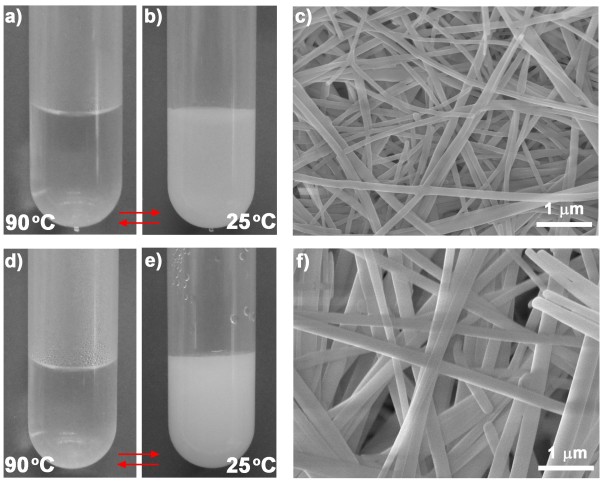
**Temperature-induced self-assembly in acetonitrile and acetonitrile-water solution. (a, b)** Photographs of FF dissolved in acetonitrile solvent at 90°C and FF assemblies formed at 25°C. **(c)** SEM image of the FF nanowires formed in acetonitrile solvent. **(d, e)** Photographs of FF dissolved in acetonitrile-water solution (95:5 *v*/*v*) at 90°C and FF assemblies formed at 25°C. **(f)** SEM image of the FF nanowires formed in acetonitrile-water solution (95:5 *v*/*v*).

To demonstrate the temperature-induced self-assembly, we chose three different solvents as the self-assembly media: acetonitrile, acetonitrile-H_2_O, and H_2_O. As shown in Figure 1, diphenylalanine peptide was dissolved in solvents at 90°C for 5 min leading to a transparent solution. When the solution was cooled to 25°C, a large number of the FF assemblies were formed during the cooling process. Figure 2a,b shows the visual change in the physical appearance from a transparent solution to white assemblies in the acetonitrile solution. The scanning electron microscopy (SEM) image showed that the assemblies consisted of uniform nanowires approximately 100 nm in diameter (Figure 2c). Following the heating-cooling procedure, the FF self-assembled into nanowires and microtubes in the acetonitrile-water solution (Figure 2d,e,f) or in water (Additional file 1: Figure S1a-c).

Interestingly, the formed nanowires or microtubes further disassembled when they were reheated to 90°C, again forming a transparent solution. During the cooling process (from 90°C to 25°C), the white FF assemblies were formed again in the solutions (Figure 2a,b,d,e and Additional file 1: Figure S1a-b). More than three heating-cooling cycles were performed to confirm the reversible self-assembly phenomenon. SEM was used to observe the resulting samples after three cycles (Additional file 1: Figure S2). The results indicated that the nanowires from both the first and third cycles had the same morphology and similar sizes. X-ray diffraction (XRD) was used to characterize the crystal structure using the FF nanowires formed in acetonitrile-H_2_O (third cycle) solution, a commonly used self-assembly medium. As shown in Additional file 1: Figure S3, the XRD spectrum was identical to that in our previous study [27], in which a hexagonal crystal structure was observed.

In addition to the temperature-induced self-assembly, HFIP was often used in previous studies to dissolve diphenylalanine peptide and then initiate the supramolecular self-assembly in aqueous or organic solvents. To verify that the reversible self-assembly behavior of FF was dependent on temperature alone, we prepared nanowires using the HFIP-initiated self-assembly method (see Additional file 1). We found that the formed FF nanowires could also again disassemble at high temperature (90°C) and self-assemble again during the cooling process (Additional file 1: Figure S1d-f). These results indicate that temperature plays a definite role in the self-assembly and disassembly of the FF peptide. Furthermore, the FF molecules self-assembled into nanostructures in the solution at low temperature (e.g., 25°C), and the resulting nanostructures then disassembled at high temperature (e.g., 90°C) again. This temperature-induced self-assembly and disassembly is perfectly reversible in different solvents.

**Figure 3:**
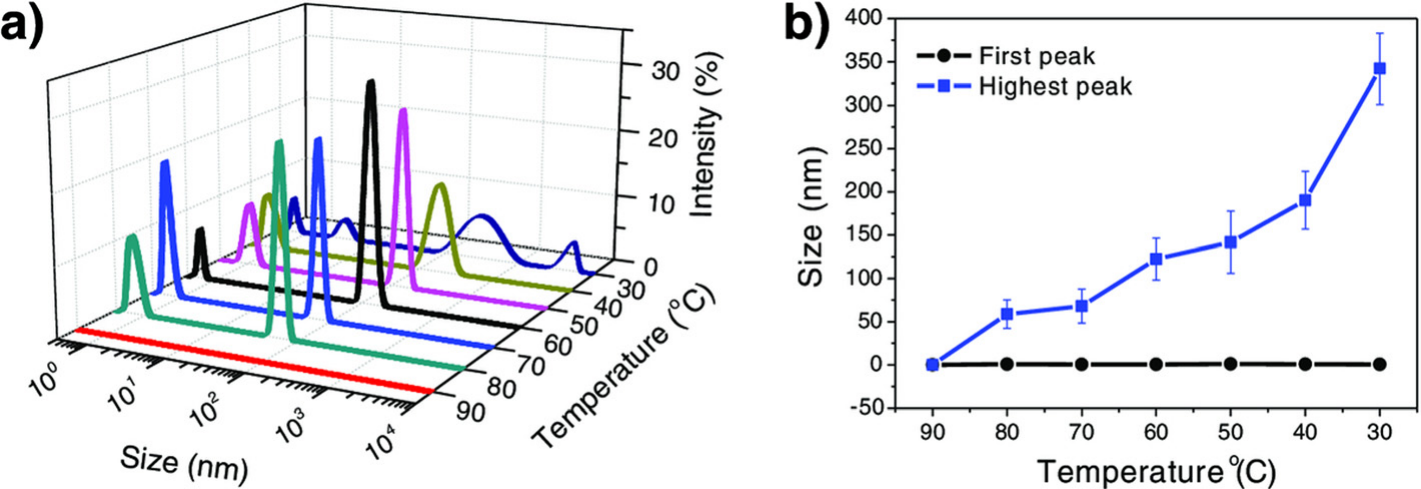
**DLS analysis. (a)** Size distribution of FF assemblies in HFIP-H_2_O solution (1:49 *v*/*v*) with different temperatures. **(b)** The size of the smallest (first peak in (a)) and the largest number of (highest peak in (a)) FF assemblies.

To better understand the temperature-induced self-assembly, dynamic light scattering (DLS) analysis was used to track the self-assembly of FF in an HFIP-H_2_O (1:49 *v*/*v*) solution during the cooling process (Figure 3). After heating the sample to 90°C, the DLS results showed no peak, suggesting that the FF was well dissolved in the solution. After decreasing the sample temperature from 90°C to 40°C, DLS curves showed two new peaks: one peak was centered at approximately 0.65 nm and exhibited no significant change, but the other peak increased gradually from 59 to 190 nm during the cooling process (Figure 3b). The results suggest that the solution may contain small FF oligomers, which further self-assembled into nanowires via a nucleation and growth process as the temperature decreases. When the temperature decreased to 30°C, larger assemblies with a broad size distribution were formed, as evidenced by the DLS curve (Figure 3a).

As we know, amino acids and some peptides exist as zwitterions in aqueous solutions. The carboxylate and amino groups can be ionized as the deprotonated (-COO^-^) and protonated (-NH_3_^+^) forms. Due to the rapid self-assembly of FF in solvents, it is difficult to determine the p*K*_a_ values. Previously, temperature has been shown to play an important role in the dissociation of -NH_3_^+^, while having less influence on -COOH [32,33]. By increasing temperature from 25°C to 90°C, the value of p*K*_2_ (-log*K*a, -NH3+) decreased, leading to a significant shift of the equilibrium to the -NH_2_ side at a fixed pH value (e.g., pH ~ 7 in our case). As a result, this dissociation equilibrium led to the change in electronic interactions and hydrogen bonding between FF-FF and FF-solvent molecules. In this case, a high solubility was achieved at 90°C. With decreasing temperature, the dissociation equilibrium shifts to the -NH_3_^+^ side. In this case, the electronic repulsion was eliminated, and the strength of hydrogen bonds (e.g., FF-FF, FF-solvent) increased, because of the enhanced hydrogen bond donor (HBD) ability of NH_3_^+^ compared to NH_2_. Meanwhile, this change in electronic interaction and hydrogen bonding was also accompanied by the enhancement of aromatic stacking between aromatic side chains, which is an important driving force for the self-assembly of FF. At 25°C, the isoelectric (p*I*) value of FF is approximately 5.5 [24]. In this study, the pH values of the solvents used for self-assembly were approximately 7, which is close to the p*I* value. The formation of FF nanowires in these solvents suggests that electrostatic and aromatic interaction may play a crucial role in the self-assembly.

**Figure 4:**
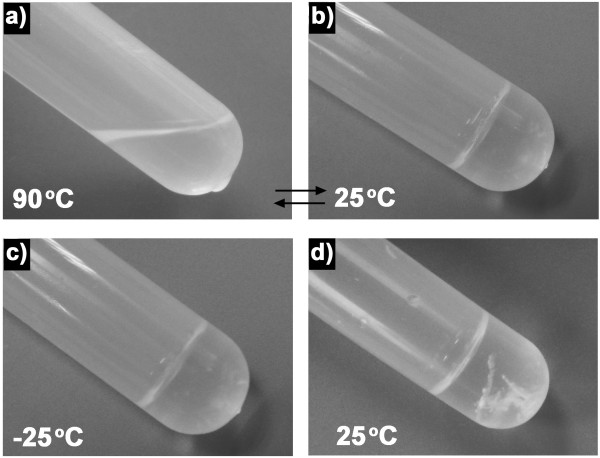
**FF solution and organogel.** Photographs of FF solution **(a)** and organogel **(b)** formed in the isopropanol solvent at 90°C and 25°C, respectively. Photographs of FF organogel stored at -25°C for 2 months **(c)** and 25°C for 3 days **(d)**.

**Figure 5:**
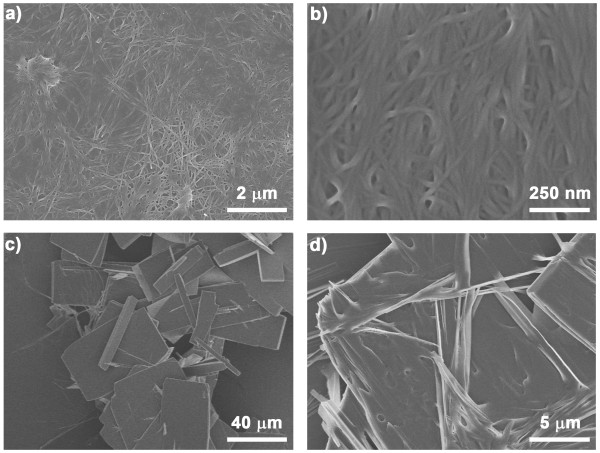
**FF organogel and flake-like aggregates.** SEM images of FF organogel **(a, b)** formed in isopropanol solvent at 25°C and flake-like aggregates **(c, d)** generated in organogel during storage at 25°C for 3 days.

We demonstrated the reversible self-assembly of the FF peptide in strongly polar solvents. Furthermore, we chose isopropanol as a weakly polar self-assembly medium. To confirm whether temperature could induce the reversible self-assembly, the FF peptide was dissolved in isopropanol at 90°C and then left at room temperature. After 30 min, a stable transparent organogel appeared. The gel was heated back up to 90°C, leading to a clear solution again within several minutes. Then, the solution was cooled to 25°C, and the organogel regenerated (Figure 4a,b). To observe the real morphology of the FF organogel, the samples were prepared by flash freezing with liquid nitrogen followed by drying under vacuum at -45°C. SEM images (Figure 5a,b) show that the organogel consists of long, flexible, non-crystalline nanofibers (no obvious characteristic peak appeared in the XRD pattern between 2*θ* of 10° and 50°, data not shown). Similar nanofibers were also observed by Yan et al. [19] in toluene and chloroform solvents. After heating and cooling for more than three cycles, the morphology of the nanofibers exhibited no change. Our results indicate a temperature-induced reversible self-assembly of the FF in isopropanol similar to that in acetonitrile and water as previously described, leading to a temperature-responsive peptide organogel.

**Figure 6:**
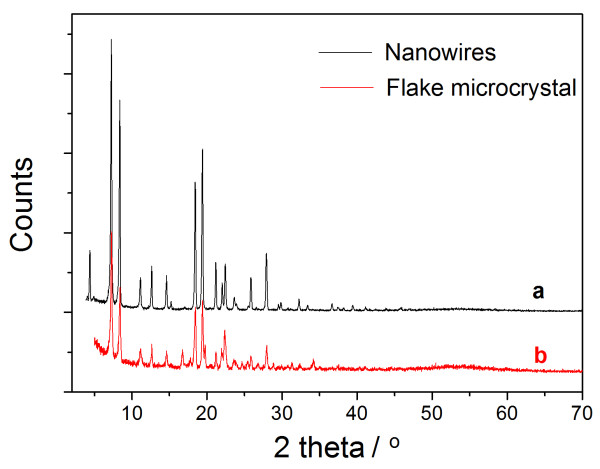
**XRD patterns.** Powder XRD patterns of the diphenylalanine nanowires formed on a glass surface **(a)** and the flake-like aggregates formed in the organogel **(b)**.

The organogel was stored at -25°C for 2 months during which no change in physical appearance (transparent) occurred, indicating high stability of the FF organogel (Figure 4c). However, when the gel was placed at 25°C for 3 days, some visible aggregates formed at the bottom of the organogel (Figure 4d). The SEM images show that the aggregates had a flake-like structure (Figure 5c,d). Such FF flakes were also previously observed in DMSO and pyridine solvents [34]. The crystalline structure of the FF flakes was also similar to that of the nanowires and microtubes formed in acetonitrile and water previously reported by our group (Figure 6) [27]. In a previous study, a polar solvent, such as ethanol, was used to induce the structural transition of FF nanofibers (gel) to microcrystals [35]. Here, we have demonstrated that temperature could also induce such structural transition. When the organogel containing flake-like aggregates was heated back up to 90°C, it formed a clear solution. After cooling to 25°C, the organogel generated again.

**Figure 7:**
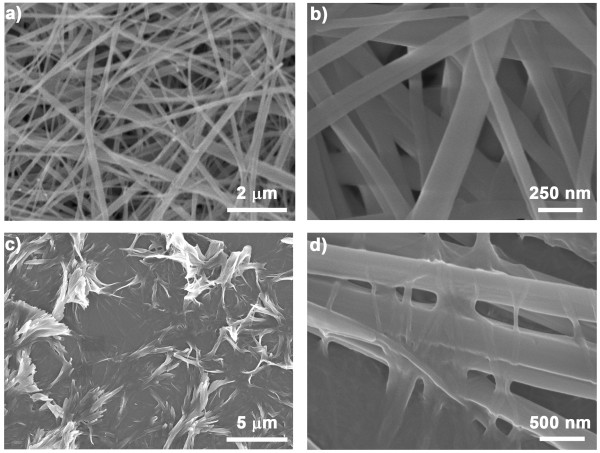
**SEM images of FF nanowires and a mixture of nanofibers and nanowires. (a, b)** SEM images of FF nanowires formed on a glass surface during the air drying of the organogel at 25°C. **(c, d)** SEM images of a mixture of nanofibers and nanowires formed on a glass surface during organogel drying at -25°C.

As demonstrated previously, the synergistic effect of the solvent and surface allows for easier control of the FF nanostructures [27]. Here, we further investigated the effect of temperature on the structural transition of the FF assemblies on a glass surface. The FF organogel was formed in isopropanol after heating at 90°C and cooling to 25°C. Afterwards, we deposited approximately 50 μL organogel on a glass surface and stored it at 25°C for air drying. Another sample was prepared by depositing approximately 50 μL organogel on a glass surface and drying at -25°C. SEM images of these two samples show that higher temperatures induce the complete structural transition of FF nanofibers to crystalline nanowires (Figure 7a,b). The crystalline structure of the nanowires was confirmed by XRD characterization. As shown in Figure 6, the nanowires were similar in crystalline structure to the flake-like aggregates, as well as the microtubes and nanowires formed in polar solvents. At a lower temperature (-25°C), some of the flexible nanofibers remained unchanged while others were transformed into rigid nanowires (Figure 7c,d). Additionally, we also prepared two samples using toluene as the self-assembly medium following the same procedure. In this case, nanofibers formed but no structural transition was observed, suggesting that the temperature-induced structural change was highly dependent on the solvent properties. Additionally, at the same temperature (25°C), the FF microcrystal generated in the organogel and the uniform nanowires formed on a glass surface. In addition to temperature effects, the coexistence of the solvent and the surface, along with the drying process, also has a significant influence on the assemblies' morphologies. However, the surface-assisted assembly mechanism still remains unclear and needs to be investigated in the future. Overall, our results demonstrate that higher temperature (e.g., 25°C) could induce the structural transition of the FF organogel (nanofibers) formed in isopropanol to the crystalline flake-like aggregates or the nanowires on a glass surface.

Organic solvents such as acetonitrile [27], tetrahydrofuran [34], ethanol [35], chloroform, and toluene [19] have been used to control the self-assembly of diphenylalanine peptide in previous works. In the crystalline FF assemblies, intermolecular hydrogen bonding exists in peptide-peptide and peptide-solvent molecules and is thought to be an essential contributor to the formation of such architectures [24]. The solvent properties that are related to the formation of hydrogen bonds such as hydrogen bond donation (HBD) ability and hydrogen bond acceptance (HBA) ability (or electron pair donation ability) should be the important determinants for the diphenylalanine self-assembly. Table 1 summarizes the property parameters of water and organic solvents [36], as well the corresponding morphology of the FF assemblies [19,27,34]. According to the morphology of the FF assemblies, the solvents are divided into three groups. Group I consists of solvents in which crystalline assemblies are formed; group II contains isopropanol in which organogel (nanofibers) and crystalline assemblies (nanowire, flake) are formed at different temperatures; and group III includes chloroform and toluene in which organogel (nanofibers) are formed. As shown in Table 1, the solvents in group I have high HBA ability or electron pair donation ability, suggesting that these solvent molecules are capable of forming hydrogen bonds with the -NH_3_^+^ or NH_2_ groups in FF molecules. This statement is validated by the corresponding hexagonal crystal structure seen in the XRD analysis [24]. Conversely, the solvents in group III have low HBD and HBA ability, which does not allow strong hydrogen bond interaction with FF molecules. In this case, the FF intermolecular interaction, such as the *π*-*π* interaction, contributes preferentially to the self-assembly of FF and leads to the formation of a stable organogel.

**Table 1:** Solvent parameters and the corresponding morphology of FF assemblies

**Group**	**Solvent**	**HBD**	**HBA**	**Morphology**
I	Water	1.17	0.47	Microtube
I	Acetonitrile	0.19	0.40	Nanowire
I	Tetrahydrofuran	0.00	0.55	Mesocrystal
I	Pyridine	0.00	0.64	Mesocrystal
II	Isopropanol	0.76	0.84	Gel/nanowire
III	Chloroform	0.20	0.10	Organogel
III	Toluene	0.00	0.10	Organogel

Interestingly, isopropanol has a high HBA ability (0.84) capable of forming hydrogen bonds with the FF molecules. According to the mechanism previously discussed, the crystalline assemblies should form in isopropanol. However, the experimental results indicate that FF preferentially self-assembled to nanofibers and organogel during the cooling process from 90°C to 25°C. This structure remained stable and unchanged at a low temperature (-25°C) and transformed into crystalline flake or nanowire at a higher temperature (e.g., 25°C). The formation of the organogel may be attributed to both the high HBD and HBA ability of isopropanol, which allows it to form intermolecular hydrogen bonds with both amino and carbonyl groups. Hydrogen bonding between isopropanol and carbonyl groups may inhibit the formation of hydrogen bonds between the FF molecules themselves, namely, head-to-tail chains (-NH_2_-H…OOC-), which is a key intermolecular interaction in the formation of crystalline structures [24,37]. Therefore, in this case, the initial FF self-assembly into an organogel was guided by other intermolecular interactions (e.g., *π*-*π* interaction). The importance of hydrogen bonds between the solvent and the carbonyl group on the supramolecular self-assembly was also demonstrated in other peptides [38].

Additionally, the HBA ability of isopropanol is slightly higher than the HBD ability (0.84 vs 0.76, Table 1). Similar to the solvents in group I, isopropanol should be capable of directing the self-assembly of the FF molecules to a crystalline structure with thermodynamic stability. We expected the self-assembly of the FF in isopropanol to be a kinetically controlled process. The nanofibers and organogel were formed by the fast self-assembly of the FF molecules guided by the strong *π*-*π* interaction. Furthermore, the nanofibers transformed to crystalline flakes or nanowires via the slow self-assembly as a result of the FF molecular rearrangement, which was guided by the hydrogen bonds between peptide-peptide and peptide-water molecules and the *π*-*π* interaction between aromatic side chains. The external energy, e.g., the high temperature, is used to overcome the energy barrier and speed up the ‘slow’ self-assembly process. Therefore, crystalline flake-like aggregates appeared in the organogel and uniform nanowires formed at 25°C on a glass surface. A similar kinetically controlled process was also found in the self-assembly of ferrocene-FF (Fc-FF), which initially self-assembled to metastable nanospheres and further reorganized to thermodynamically stable nanofibers with the introduction of a mechanical force [39]. In this study, the temperature-induced structural transition from an organogel (nanofibers) to crystalline flakes and nanowires was initially found for the FF self-assembly system.

## Conclusions

In summary, we demonstrated the temperature-triggered reversible self-assembly of diphenylalanine peptide into microtubes, nanowires, and an organogel in different solvents. In polar solvents, the dissociation equilibrium of the FF molecules was highly dependent on temperature, which likely led to the change in the electronic and aromatic interactions between the FF molecules themselves and induced the reversible self-assembly. Furthermore, we demonstrated the temperature-induced structural transition of an organogel in isopropanol to crystalline flakes and nanowires. We infer that self-assembly of the FF in isopropanol with high HBD and HBA ability, unlike in chloroform/toluene (low HBD and HBA ability), is a kinetically controlled process. The nanofibers and organogel were preferentially formed by the fast self-assembly of FF molecules guided by a strong *π*-*π* interaction and transformed to crystalline structures by introducing external energy (e.g., increased temperature) to overcome the energy barrier.

## Competing interests

The authors declare that they have no competing interests.

## Authors’ contributions

RLH, YFW, and WQ designed the research. RLH and YFW performed the research and contributed equally to this work. RLH, YFW, WQ, RXS, and ZMH analyzed the data and wrote the paper. All authors read and approved the final manuscript.

## Supplementary Material

Additional file 1 **Supporting information.** A document showing supplementary experiments and figures. Click here for file
