# Supplementary material for: Temperature-induced reversible self-assembly of diphenylalanine peptide and the structural transition from organogel to crystalline nanowires
Source: Nanoscale Res Lett. 2014 Dec 3;9(1):653. doi: 10.1186/1556-276X-9-653 (PMC4266524; doi:10.1186/1556-276X-9-653)
Supplement: Additional file 1 — Supporting information. A document showing supplementary experiments and figures. [file 1556-276X-9-653-S1.doc]

**Addtional file 1**

**Temperature-induced reversible self-assembly of diphenylalanine peptide and the structural transition from organogel to crystalline nanowires**

**Renliang Huang ^1,2^, Yuefei Wang ^2^, Wei Qi ^2,3*^,** **Rongxin Su ^2,3^ and** **Zhimin He^2^**

^1^ School of Environmental Science and Engineering, Tianjin University, Tianjin 300072, P. R. China;

^2^ State Key Laboratory of Chemical Engineering, School of Chemical Engineering and Technology, Tianjin University, Tianjin 300072, P. R. China;

^3^ Collaborative Innovation Center of Chemical Science and Engineering (Tianjin), Tianjin 300072, P. R. China

Correspondence: [qiwei@tju.edu.cn](mailto:qiwei@tju.edu.cn); Tel: +86 22 27407799

**Supplementary experiments**

**HFIP-initiated self-assembly of FF into nanowires in water** The diphenylalanine stock solution was freshly prepared by dissolving the peptides in HFIP at a concentration of 100 mg mL^-1^ (prepared just before use). The FF peptide stock solution was then diluted to a final concentration of 2 mg mL^-1^ in water. The resulting solution was shaken for several seconds, and then aged at room temperature for 2 h without disturbance.

**Supplementary Figures**


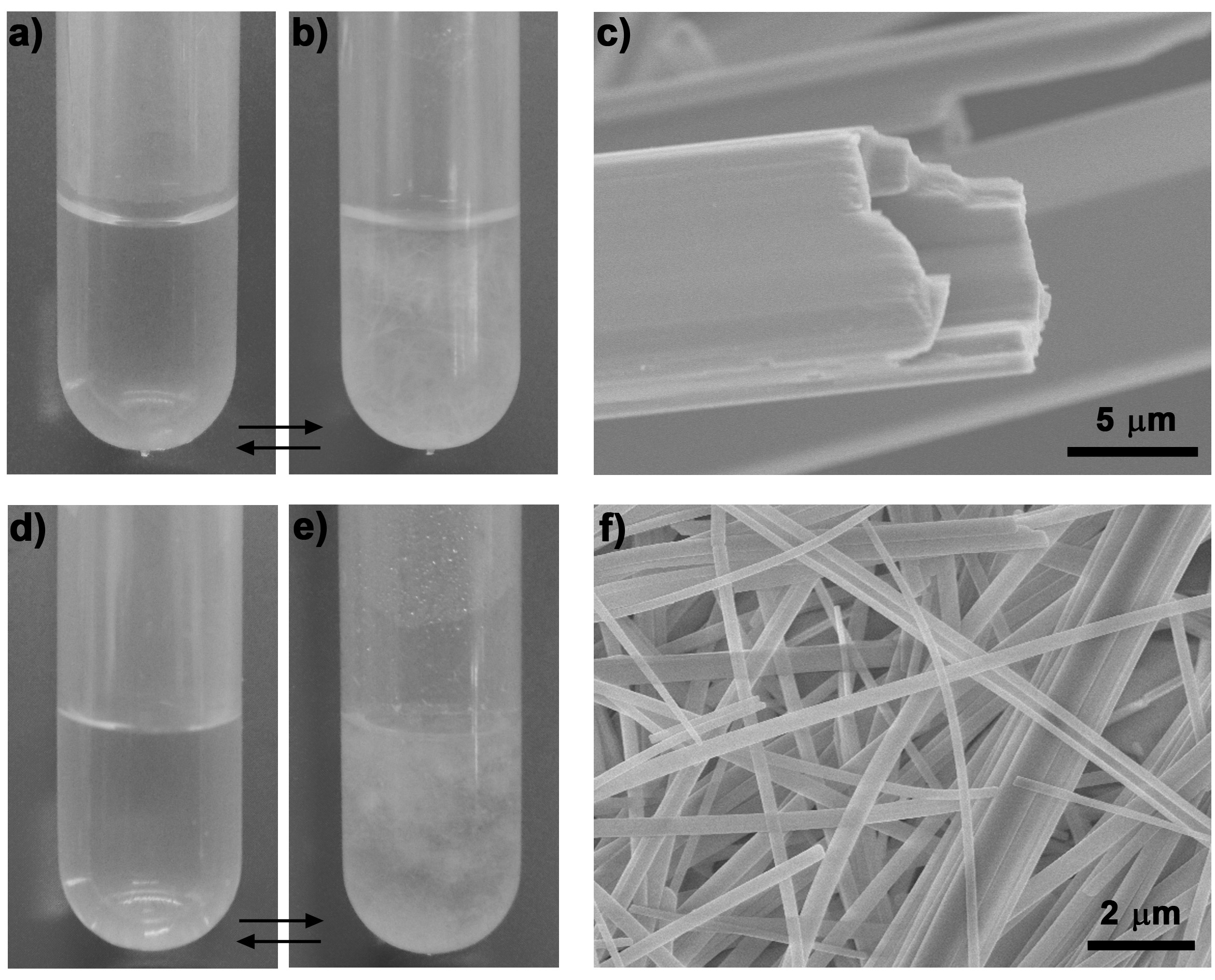


**Additional file 1: Figure S1** a-b) Photographs of FF dissolved in water at 90 ^o^C and FF assemblies formed at 25 ^o^C. c) SEM image of the FF microtube formed in water. d-e) Photographs of FF dissolved in HFIP-water (1:49 V/V) solution at 90 ^o^C and FF assemblies formed at 25 ^o^C. f) SEM image of the FF nanowires formed in HFIP-water solution


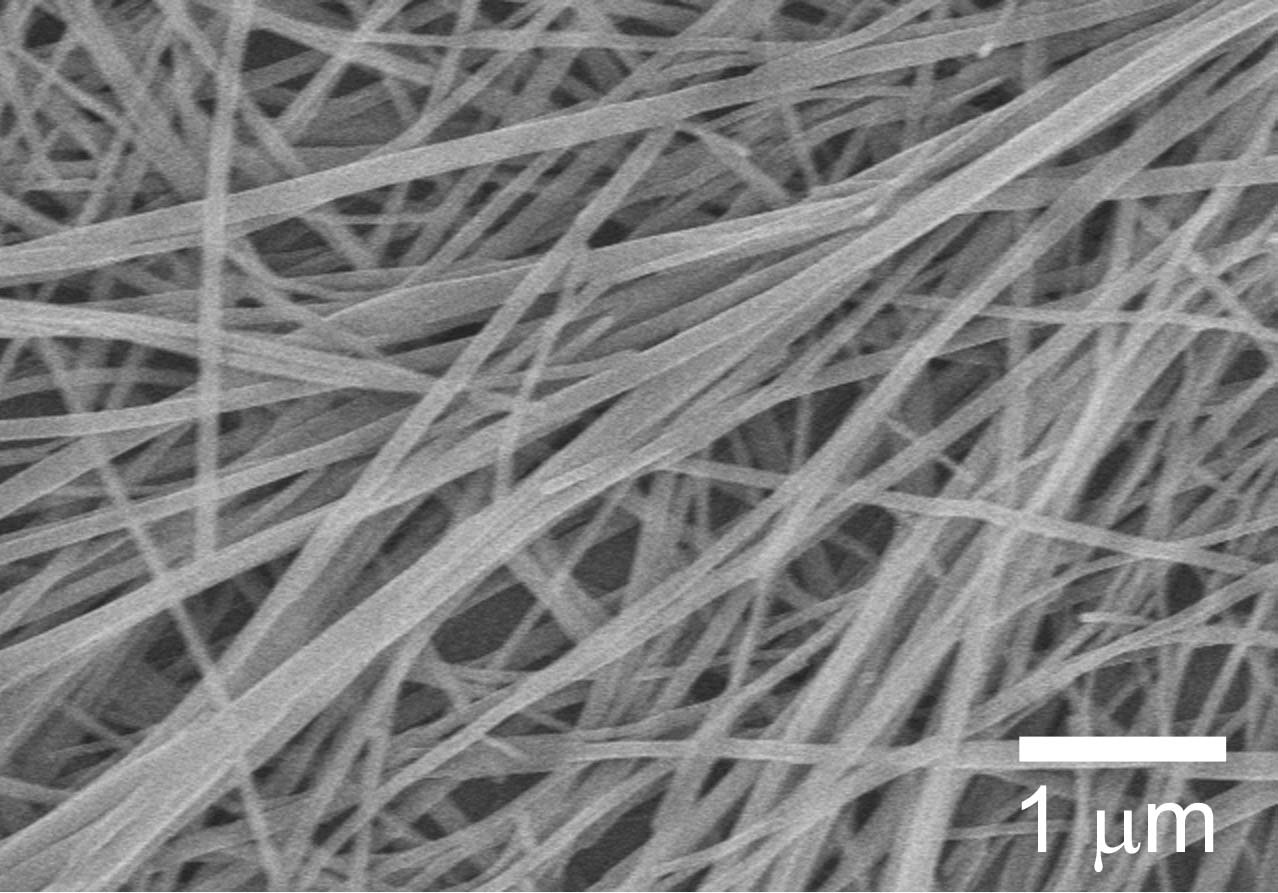

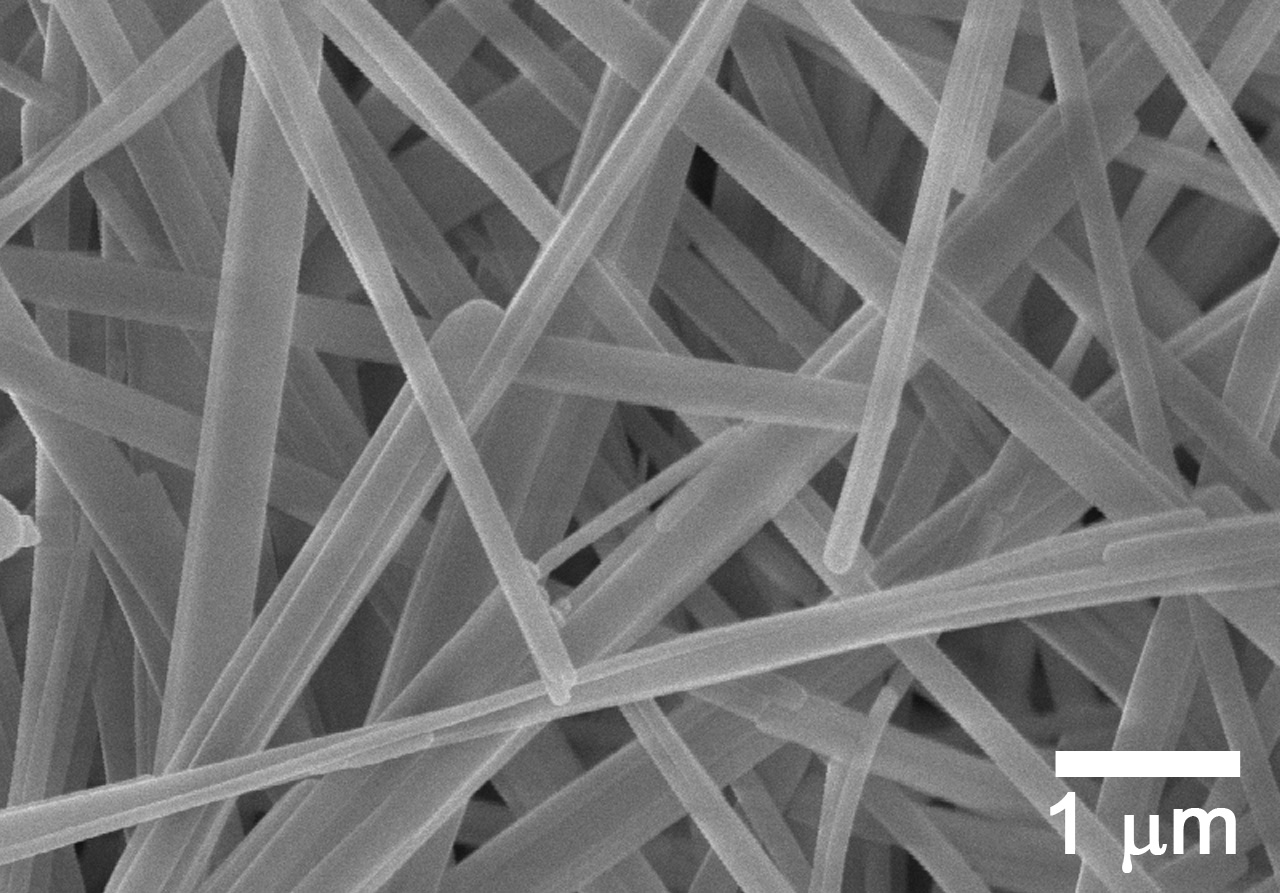


**a)**

**b)**

**Additional file 1: Figure S2** SEM images of nanowires after heating (90 ^o^C)-cooling (25 ^o^C) for three cycles in acetonitrile (a) and acetonitrile-H_2_O (b).

**Additional file 1: Figure S3** Powder XRD patterns of the diphenylalanine nanowires formed in acetonitrile-H_2_O solution at 25 ^o^C.
